# Impact of thoracic radiotherapy on first‐line treatment outcomes in ES‐SCLC patients

**DOI:** 10.1002/cam4.70175

**Published:** 2024-09-06

**Authors:** Xiaoli Mu, Yixin Zhou, Qing Liu, Jiantao Wang, Feng Xu, Feng Luo, Ke Wang, Lu Li, Panwen Tian, Yalun Li, Jiewei Liu, Yan Zhang, Jiyan Liu, Yan Li

**Affiliations:** ^1^ The Department of Biotherapy, Cancer Center West China Hospital, Sichuan University Chengdu Sichuan China; ^2^ Lung cancer center West China Hospital, Sichuan University Chengdu Sichuan China

**Keywords:** Chemotherapy, Extensive‐stage small cell lung cancer, Immunotherapy, Survival outcomes, Thoracic radiotherapy

## Abstract

**Background:**

The therapeutic advantage of thoracic radiotherapy (tRT) as an adjunct to first‐line immunotherapy and chemotherapy in patients with extensive‐stage small cell lung cancer (ES‐SCLC) remains unclear. We sought to elucidate this in a retrospective cohort study comparing the effectiveness and safety of tRT in combination with first‐line immunotherapy and chemotherapy.

**Methods:**

Our retrospective study included patients with ES‐SCLC, treated at the West China Hospital between January 2019 and December 2022. They received first‐line immunotherapy and chemotherapy and were categorized into two cohorts based on the administration of tRT. The primary outcomes were overall survival (OS) and progression‐free survival (PFS). Cox regression analysis was utilized to identify potential independent predictors of prognosis and to compare the treatment outcomes across various patient subgroups. Treatment‐related toxicities across both cohorts were compared using the Chi‐squared test.

**Results:**

A total of 99patients were eligible for the study, out of which 55 received tRT. The medianduration of follow‐up was 39 months. Remarkably, patients who received tRTdemonstrated superior OS and PFS in comparison to those who did not (*P* < 0.05). Subgroup analysis further confirmed these findings. Multivariate analysisidentified treatment group and liver metastasis as independent prognosticfactors (*P* < 0.05). The incidence of grade 3‐4 adverse events showed nostatistically significant difference between the two cohorts.

**Conclusions:**

Thus, weconfirmed that the addition of tRT to the conventional regimen of first‐linechemotherapy and immunotherapy yields better survival outcomes without asignificant increase in toxicity.

## Graphical abstract

Small cell lung cancer (SCLC) is a highly aggressive malignancy associated with a bleak prognosis. Combination of immunotherapy and chemotherapy has become the preferred first‐line treatment option for ES‐SCLC. Addition of tRT to the conventional regimen of first‐line chemotherapy and immunotherapy yields better survival outcomes without a significant increase in toxicity.

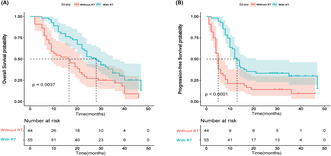

## 1 INTRODUCTION

Small cell lung cancer (SCLC) is an aggressive form of lung cancer that accounts for approximately 15% of all lung cancer cases.^1^, ^2^ Originating from neuroendocrine cells, it is often poorly differentiated and associated with a high mortality rate; around 250,000 individuals globally succumb to this disease each year.^3^, ^4^ Hallmarks of SCLC include rapid progression, early propensity for metastasis, resistance to treatments, and high recurrence rate.^5^, ^6^ According to the Veterans Lung Cancer Association (VALG) stage II classification, SCLC is divided into limited stage (LS‐SCLC) and extensive stage (ES‐SCLC). Unfortunately, approximately 70% of SCLC patients present with ES‐SCLC at the time of initial diagnosis, which accounts for the grim 5‐year overall survival (OS) rate of less than 7%.^4^, ^7^

Historically, the standard first‐line treatment for ES‐SCLC has been a combination of etoposide with either cisplatin (EP) or carboplatin (EC). Moreover, in recent years, immune checkpoint inhibitors (ICIs) targeting PD‐1 and PD‐L1 have demonstrated considerable potential in treating SCLC. As evidenced by the IMpower133 and CASPIAN randomized trials, adding atezolizumab or durvalumab to front‐line chemotherapy with EP/EC significantly enhances survival times compared to chemotherapy alone.^8^, ^9^ Thus, a combination of immunotherapy and chemotherapy has become the preferred first‐line treatment option for ES‐SCLC.^10^

Radiotherapy stands as one of the primary treatment modalities for malignancies causing locoregional damage and is deemed a crucial element in SCLC management.^11^ Particularly, patients with ES‐SCLC who respond well to first‐line chemotherapy may reap additional benefits from thoracic radiotherapy (tRT), especially those with residual thoracic lesions and minor distant metastatic lesions.^12^ Past research has shown that the low‐dose chest radiotherapy mitigates the risk of symptomatic chest recurrence and may extend survival for a subset of ES‐SCLC patients.^13^, ^14^ Furthermore, recent findings indicate that the combination of ICIs and radiotherapy may engender a synergistic effect, enhancing antitumor efficacy.^15^, ^16^ This suggests that the combination of ICIs and radiotherapy could serve as a promising therapeutic strategy. Corroborating this notion, a phase 1/2 clinical trial by Welsh et al. displayed that the integration of pembrolizumab with concurrent chemoradiation therapy for LS‐SCLC was both safe and effective, with encouraging results.^17^ Yet, the question remains whether tRT can enhance the effects of immunotherapy and extend the survival time of patients with ES‐SCLC.

In the current study, we aim to explore the potential benefits of tRT for patients with ES‐SCLC undergoing first‐line treatment with immunotherapy and chemotherapy.

## 2 METHODS

### 2.1 Patients

**FIGURE 1:**
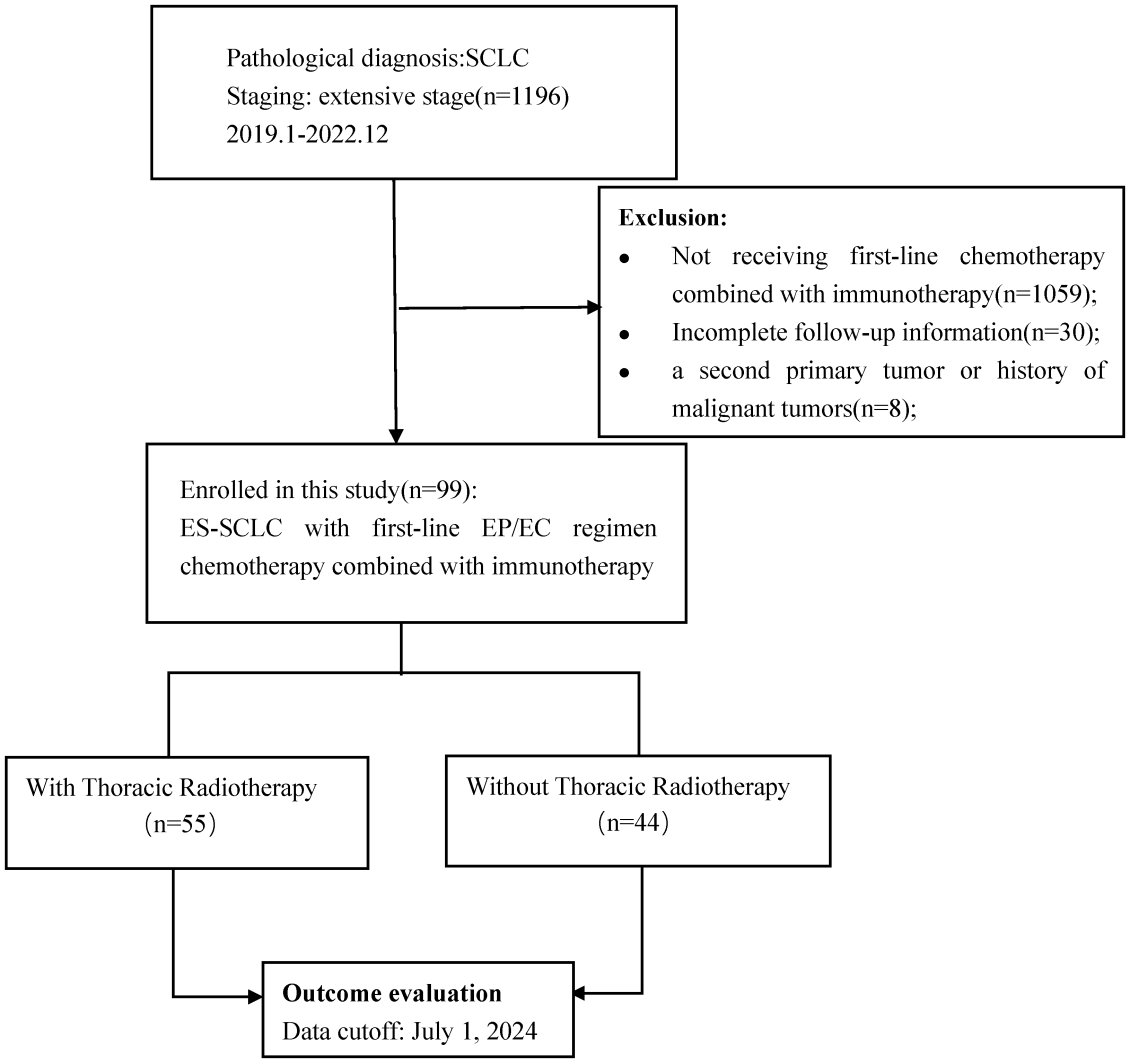
Patient selection flow chart.

We conducted a retrospective analysis of patients with ES‐SCLC who were treated at West China Hospital between January 2019 and December 2022. The study included patients who met the following criteria: (a) a cytologic or pathologic diagnosis confirming SCLC; (b) extensive‐stage disease as classified by the VALG staging; (c) administration of immunotherapy plus EP/EC‐based chemotherapy as first‐line treatment; and (d) availability of complete clinical follow‐up information. Exclusion criteria encompassed: (a) patients with limited‐stage disease; (b) patients who did not receive immunotherapy plus EP/EC‐based chemotherapy as first‐line treatment; (c) patients with missing or incomplete data; and (d) patients with a previous history of malignancy or a second primary tumor. This study was approved by the Institutional Review Board of West China hospital of Sichuan University (No. 2023‐1547). Figure 1 provides a flowchart of patient selection.

### 2.2 Treatment regimen

All participants received EP/EC‐based chemotherapy in conjunction with immunotherapy as their first‐line treatment. Etoposide was administered intravenously at a dosage of 80–100 mg/m^2^ on days 1–3 of each 21‐day cycle. Cisplatin was given intravenously at a dosage of 60–80 mg/m^2^ on day 1 of each 21‐day cycle, or carboplatin was administered intravenously at an area under the curve (AUC) of 5–6 on day 1 of each 21‐day cycle. The selection between cisplatin and carboplatin was made based on individual patient characteristics and medical history, in consultation with a qualified healthcare provider. Durvalumab was given intravenously at a dose of 1500 mg every 4 weeks, and atezolizumab was administered intravenously at a dose of 1200 mg every 3 weeks. Other immunotherapy drugs, such as the PD‐1 inhibitor pembrolizumab, were administered intravenously at a dose of 200 mg every 3 weeks. The duration of treatment was tailored to each patient's response and tolerance to therapy, typically continuing until disease progression or the manifestation of unacceptable toxicity.

All patients with ES‐SCLC who completed first‐line immunotherapy and chemotherapy underwent a thorough evaluation to determine their eligibility for tRT. This included assessment of disease extension after chemoimmunotherapy, Eastern Cooperative Oncology Group performance status (ECOG PS) 0–2, adequate organ function, and measurable or evaluable disease on imaging. Additionally, other factors such as age, comorbidities, and patient preferences were considered in the decision‐making process for tRT. The timing of tRT administration varied based on individual patient factors and treatment response. Typically, tRT was initiated within 4–6 weeks after completion of first‐line immunotherapy and chemotherapy, aiming to optimize treatment outcomes while minimizing treatment delays and ensuring adequate recovery from initial systemic therapy. Treatment planning techniques, target delineation, planned target volume (PTV) margin expansion, dose prescription, and fractionation were determined by the attending radiation oncologist. Radiotherapy doses were based on a comprehensive assessment of each patient's condition. The tRT protocol for ES‐SCLC patients ranged from 14 Gy/7 f to 66 Gy/30 f, allowing for tailored treatment intensity based on individual factors, disease stage, and treatment goals. Lower doses, like 14 Gy/7 f, may serve as palliative treatments aiming for symptomatic relief or local control in frail patients or those unable to tolerate more aggressive regimens, while higher doses, such as 66 Gy/30 f, could be used for consolidation post‐chemotherapy or as part of definitive treatment. In terms of radiotherapy techniques, patients received three‐dimensional conformal radiotherapy (3D‐CRT) or intensity‐modulated radiotherapy based on CT localization, including (but not limited to) 4D‐CT and/or PET‐CT simulation. Patients underwent one fraction of radiation therapy per day, five times per week.

### 2.3 Outcomes

The primary outcomes of this study were OS and progression‐free survival (PFS). OS was defined as the period from the start of treatment to death from any cause. PFS was defined as the interval from the start of treatment to either disease progression or death from any cause. Response Evaluation Criteria in Solid Tumors (RECIST) version 1.1 was used to assess tumor response in our study. Therapy‐related toxicities were evaluated using the Common Terminology Criteria for Adverse Events (CTCAE) version 4.0.

### 2.4 Statistical analysis

Comparisons of baseline characteristics and therapy‐related toxicities across different treatment groups were performed using the Chi‐squared test. Clinical outcomes, such as OS and PFS, were examined and compared using Kaplan–Meier curves and log‐rank tests. Independent prognostic factors were identified using univariate and multivariate Cox proportional hazards analyses. Statistical analyses were performed using R version 3.62 and IBM SPSS 25.0 (Armonk, NY, USA). All statistical tests were two‐sided, and a *p* value <0.05 was deemed significant.

## 3 RESULTS

### 3.1 Patient characteristics

This study included 99 eligible patients. Within this cohort, 44 patients were treated with a combination of first‐line chemotherapy and immunotherapy, while the remaining 55 were administered tRT in addition to this regimen. The cohort's median age was 64 years, with the majority being males (84.8%) and with a history of smoking. Patients diagnosed with stage IVa and stage IVb disease constituted 62.6% and 37.4% of the cohort, respectively. There was a trend towards fewer patients with stage IVB in the radiotherapy group. However, baseline characteristics were comparable (*p* = 0.14).The majority of tumors were located in the right lung (60.6%). There was one (1%) patient with an ECOG PS score of 2. Prior to first‐line treatment, 16 patients presented with brain metastases and 32 with liver metastases. Most patients demonstrated abnormal neuron‐specific enolase (NSE) levels before treatment. A total of 45 patients were treated with the EP regimen, and 54 with the EC regimen. Furthermore, 33 patients received more than four cycles of chemotherapy. Among the 66 patients who received four or fewer cycles, three had to discontinue after three cycles due to intolerable chemotherapy‐related toxicities. Additionally, 12 of the included patients had received prophylactic intracranial radiation therapy. Concerning immunotherapy, 50 patients (50.5%) were administered durvalumab, 37 (37.4%) received atezolizumab, and the remaining 12 (12.1%) were treated with other PD‐1 inhibitors. The baseline clinical characteristics of each group are summarized in Table 1.

**TABLE 1:** Clinicopathological characteristics of patients with ES‐SCLC at baseline.

Characteristics	Total *N* = 99	With RT *N* = 55	Without RT *N* = 44	*p* value*
Age				
≤60	40 (40.4%)	24 (43.6%)	16 (36.4%)	0.46
>60	59 (59.6%)	31 (56.4%)	28 (63.6%)	
Sex				
Male	84 (84.8%)	46 (83.6%)	38 (86.4%)	0.71
Female	15 (15.2%)	9 (16.4%)	6 (13.6%)	
Smoking history				
Current/former smokers	71 (71.7%)	40 (72.73%)	31 (70.45%)	0.80
Never smokers	28 (28.3%)	15 (27.27%)	13 (29.55%)	
PS				
0	83 (83.8%)	48 (87.27%)	35 (79.55%)	0.29
1	15 (15.2%)	6 (10.91%)	9 (20.45%)	
2	1 (1.0%)	1 (1.82%)	0	
NSE				
0–20 ng/ml	11 (11.1%)	9 (16.36%)	2 (4.55%)	0.06
>20 ng/ml	88 (88.9%)	46 (83.64%)	42 (95.45%)	
Tumor site				
Left	39 (39.4%)	18 (32.73%)	21 (47.73%)	0.13
Right	60 (60.6%)	37 (67.27%)	23 (52.27%)	
Stage				
IVa	62 (62.6%)	38 (69.09%)	24 (54.55%)	0.14
IVb	37 (37.4%)	17 (30.91%)	20 (45.45%)	
Chemotherapy regimens				
EP	45 (45.5%)	27 (49.1%)	18 (40.9%)	0.42
EC	54 (54.5%)	28 (50.9%)	26 (59.1%)	
Chemotherapy cycles				
≤4	66 (66.7%)	33 (60%)	33 (75%)	0.12
>4	33 (33.3%)	22 (40%)	11 (25%)	
Immunotherapy				
Atezolizumab	37 (37.4%)	20 (36.4%)	17 (38.6%)	0.96
Durvalumab	50 (50.5%)	28 (50.9%)	22 (50%)	
Other(PD‐1)	12 (12.1%)	7 (12.7%)	5 (11.4%)	
Brain metastasis				
Yes	16 (16.2%)	7 (12.7%)	9 (20.5%)	0.30
No	83 (83.8%)	48 (87.3%)	35 (79.5%)	
Liver metastases				
Yes	32 (32.3%)	14 (25.45%)	18 (40.9%)	0.10
No	67 (67.7%)	41 (74.55%)	26 (59.1%)	
Propylactic intracranial irradiation				
Yes	12 (12.1%)	10 (18.2%)	2 (4.5%)	0.86
No	87 (87.9%)	45 (81.8%)	42 (95.5%)	
Best response				
Objective responsers	67 (67.7%)	42 (76.3%)	25 (56.8%)	0.04

* *p* > 0.05.

### 3.2 Treatment outcomes

**FIGURE 2:**
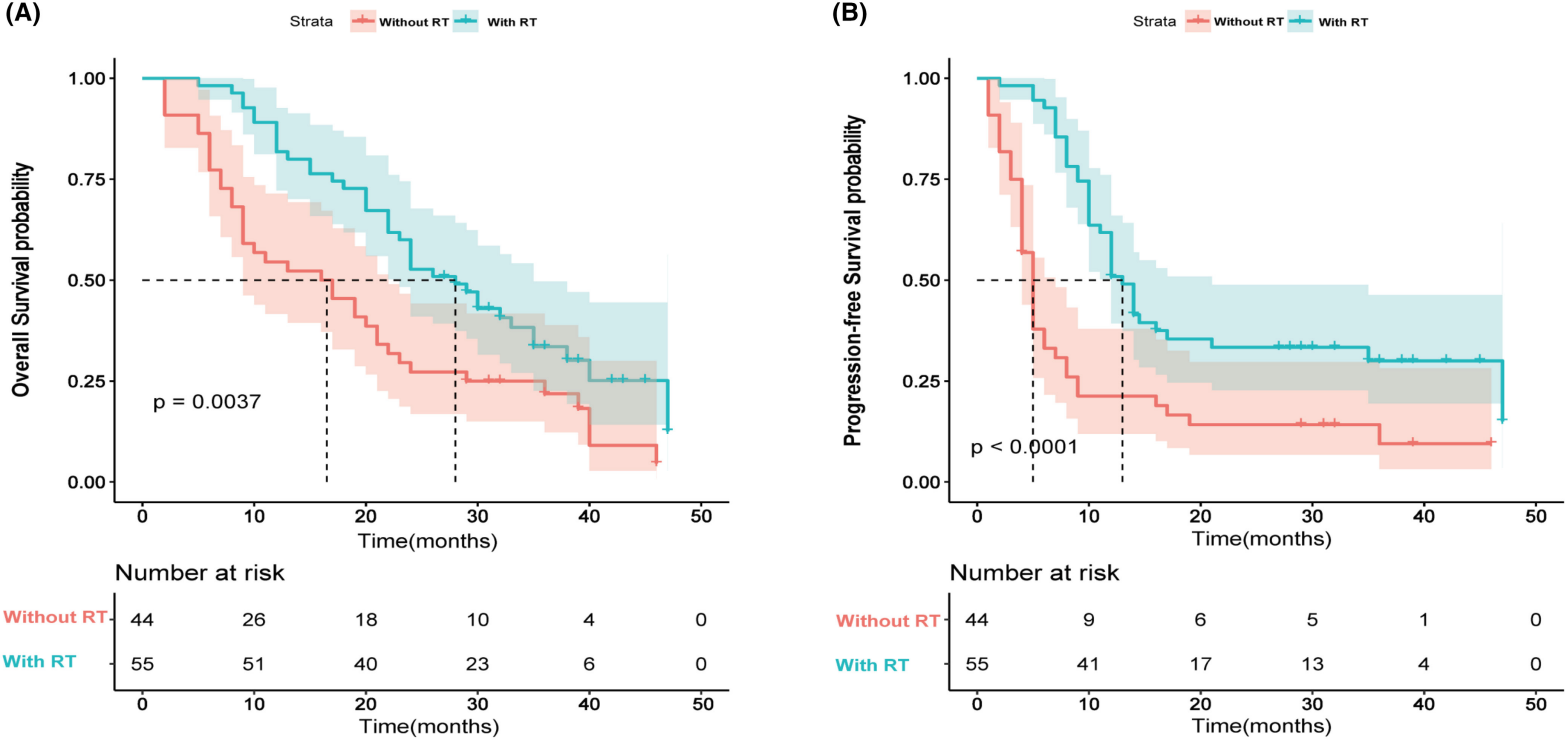
Kaplan–Meier(KM) curves of overall survival (A), progression‐free survival (B) in ES‐SCLC patients treated with tRT or without tRT.

**FIGURE 3:**
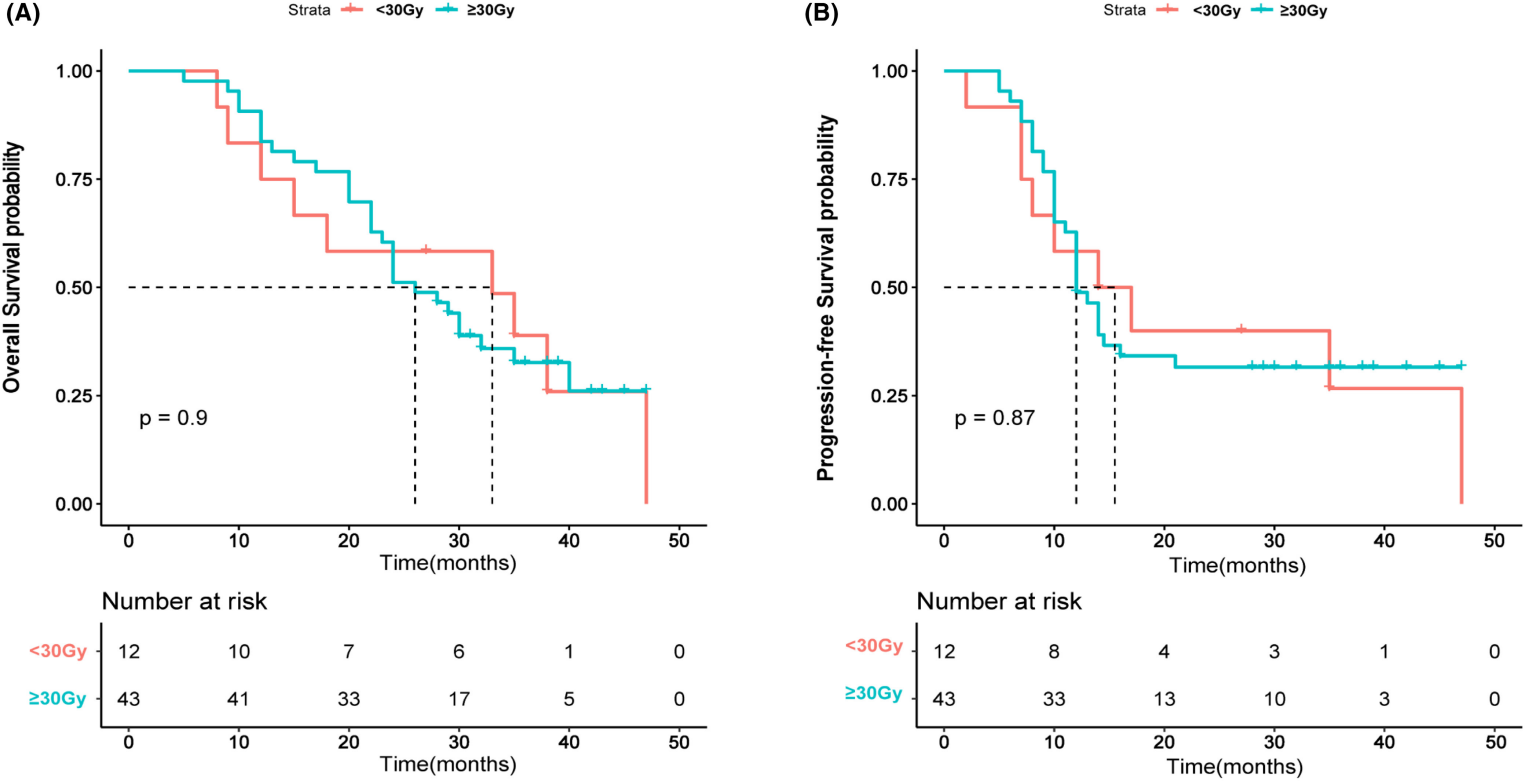
KM curves for overall survival (A) and progression‐free survival (B) of ES‐SCLC patients treated with different radiation doses.

**FIGURE 4:**
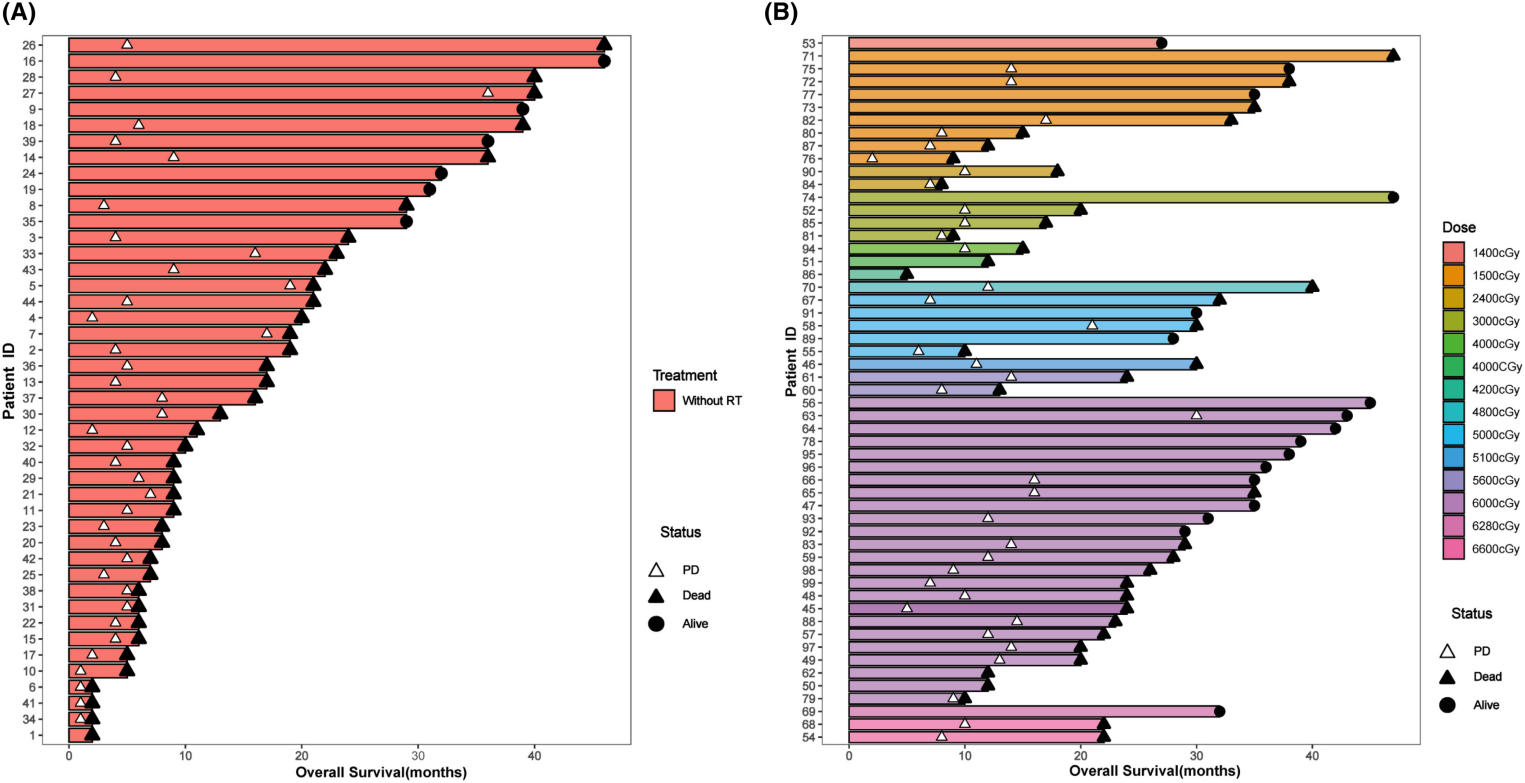
Swimmer plot of overall survival for patients who did not receive(A) or received(B) thoracic radiation therapy.

The median follow‐up time for this study was 39 months. During this period, 38 patients (86.4%) from the non‐tRT group and 38 patients (69.1%) from the tRT group died. The median OS for the entire cohort was 22 months, and the median PFS was 10 months. Efficacy evaluations revealed that 67 patients achieved a partial response, comprised of 42 tRT‐treated and 25 non‐tRT‐treated patients. The objective response rates (ORR) for these two groups were 76.3% and 56.8%, respectively. Patients who underwent tRT demonstrated a significant improvement in both OS and PFS compared to those who did not. These results were corroborated by a Kaplan–Meier analysis of OS and PFS (Figure 2). Results from this study suggested that tRT could be associated with a statistically significant improvement in both median OS (28 months) and PFS (13 months) when compared to the group that did not receive tRT (median OS: 16 months; median PFS: 5 months). Further, a Kaplan–Meier analysis was conducted to explore the influence of radiotherapy dose on patient prognosis. Patients were divided into two groups based on their radiotherapy dose: those who received less than 30 Gy and those who received 30 Gy or more. This cutoff was chosen to distinguish between lower doses often used for palliative purposes (less than 30 Gy), aimed at symptom relief and local control, and higher doses (30 Gy or more), which are typically used for consolidation or curative intent following chemotherapy. No significant difference in OS or PFS between these two groups was observed in the analysis (Figure 3). As our study was retrospective, radiotherapy doses varied according to individual clinical situations and the judgment of the treating physicians. To provide a clearer understanding, we have included a swimmer plot (Figure 4) that illustrates the dose distribution among patients and their corresponding OS. Figure 4A illustrates the OS rates and disease progression for patients in the non‐tRT group, where 6 out of 44 patients were alive at the time of data cut‐off. Conversely, Figure 4B displays a swimmer plot of OS for patients who received tRT, with radiotherapy doses ranging from 1400 to 6600 cGy, and 17 out of 55 patients were alive at the data follow‐up cut‐off.

### 3.3 Prognostic analysis

A Cox proportional hazards regression model was applied for the univariate analysis, as shown in Table 2. This analysis suggested that factors including liver metastases, brain metastases, and the treatment group (with or without tRT) held associations with patient prognosis (*p* < 0.1). Notably, while staging was identified as a significant prognostic factor for PFS in univariate analysis, it did not emerge as an independent prognostic factor for OS. These factors were subsequently included in the multivariate analysis. The multivariate analysis (Table 3) identified the treatment group and liver metastases as independent predictors of both PFS and OS (*p* < 0.05).

**TABLE 2:** Univariate Analyses of Factors Predicting OS and PFS Outcomes.

Univariate analysis	OS		PFS	
Variables	HR (95%CI)	*p* value*	HR (95%CI)	*p* value*
Age				
≤60	Reference		Reference	
>60	1.152 (0.727–1.825)	0.546	0.872 (0.550–1.383)	0.561
Sex				
Male	Reference		Reference	
Female	1.360 (0.732–2.528)	0.331	1.278 (0.688–2.375)	0.438
Smoking history				
Never smokers	Reference		Reference	
Current/former smokers	0.804 (0.492–1.316)	0.386	0.714 (0.436–1.169)	0.180
PS				
0	Reference		Reference	
1	1.506 (0.599–2.078)	0.731	1.074 (0.577–2.001)	0.821
2	0.780 (0.108–5.654)	0.806	0.936 (0.127–6.668)	0.936
Cycle				
≤4	Reference		Reference	
>4	0.794 (0.493–1.278)	0.342	0.754 (0.468–1.212)	0.243
Site				
Left	Reference		Reference	
Right	1.070 (0.676–1.692)	0.773	0.979 (0.618–1.551)	0.929
Stage				
IVa	Reference		Reference	
IVb	1.434 (0.905–2.274)	0.125	1.589 (1.002–2.517)	0.049
Brain metastasis				
No	Reference		Reference	
Yes	1.997 (1.107–3.604)	0.022	1.834 (1.022–3.294)	0.042
Liver metastasis				
No	Reference		Reference	
Yes	2.470 (1.551–3.934)	0.001	2.730 (1.694–4.400)	0.001
Group				
With RT	Reference		Reference	
Without RT	1.914 (1.214–3.018)	0.005	2.653 (1.672–4.209)	0.001
NSE				
0–20 ng/ml	Reference		Reference	
>20 ng/ml	1.456 (0.698–3.036)	0.316	1.485 (0.713–3.094)	0.291
Chemotherapy regimens				
EP	Reference		Reference	
EC	1.433 (0.908–2.262)	0.123	1.017 (0.646–1.602)	0.941
Propylactic intracranial irradiation				
No	Reference	0.380	Reference	
Yes	0.731 (0.363–1.471)		0.784 (0.391–1.575)	0.495
Immunotherapy				
Atezolizumab	Reference		Reference	
Durvalumab	0.752 (0.459–1.232)	0.258	0.739 (0.452–1.210)	0.315
Other(PD‐1)	1.385 (0.692–2.770)	0.358	2.288 (1.121–4.673)	0.023

* *p* < 0.1.

**TABLE 3:** Multivariate Analyses of Factors Predicting OS and PFS Outcomes.

Multivariate analysis	OS		PFS	
Variables	HR (95% CI)	*p* value	HR (95% CI)	*p* value*
Liver metastasis		0.002		0.001
No	Reference		Reference	
Yes	2.115 (1.312‐3.410)		2.489 (1.464‐4.232)	
Brain metastasis		0.129		0.826
No	Reference		Reference	
Yes	1.592 (0.874‐2.901)		1.077 (0.556‐2.085)	
Group		0.030		0.001
With RT	Reference		Reference	
Without RT	1.670 (1.051‐2.652)		2.486 (1.547‐3.998)	
Stage		/		0.818
IVa	/		Reference	
IVb	/		1.066 (0.617‐1.841)	

* *p* < 0.05.

**FIGURE 5:**
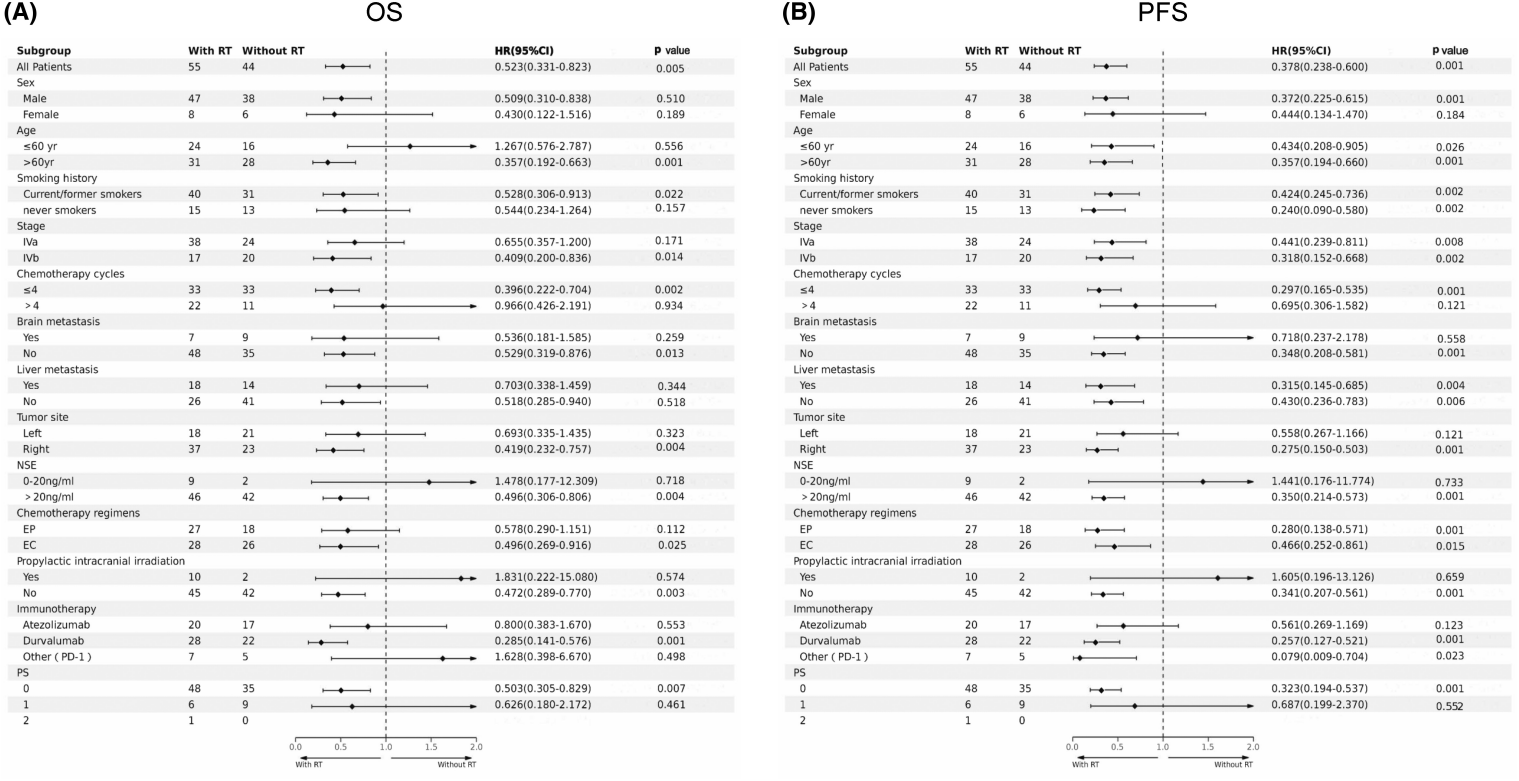
Forest plots for each subgroup analysis. (A) Forest plot for Overall Survival (OS). (B) Forest plot for Progression‐Free Survival (PFS).

To better understand the survival differences between the two treatment modalities for patients with different risk levels, we conducted a subgroup stratification analysis. For the majority of stratification factors, patients who underwent tRT demonstrated significantly better OS and PFS compared to those who did not (Figure 5), supporting the potential benefit of tRT in ES‐SCLC management.

### 3.4 Treatment‐related adverse events

The most commonly observed treatment‐related adverse events (AEs) in both groups were neutropenia and leukopenia, with incidences of 18.18% (10 patients) in the tRT group and 6.82% (3 patients) in the non‐tRT group. During the treatment period, no significant difference was observed in the occurrence of severe (grade 3–4) AEs between the two groups, according to statistical analysis (Table 4). Of note, two patients in the tRT group developed grade 3 radiation pneumonitis. After prompt intervention, these patients' symptoms were alleviated, and both were alive at the data cut‐off.

**TABLE 4:** Grade 3–4 toxic reactions during treatment in two groups of ES‐SCLC patients.

Toxicities	Total *N* = 99	With RT *N* = 55	Without RT *N* = 44	*p* value*
Neutropenia	13 (13.13%)	10 (18.18%)	3 (6.82%)	0.096
Leucopenia	13 (13.13%)	10 (10.91%)	3 (6.82%)	0.096
Thrombocytopenia	3 (3.03%)	3 (5.45%)	1 (2.27%)	0.4243
Anemia	7 (7.07%)	4 (7.27%)	3 (6.82%)	0.9301
Nausea	5 (5.05%)	3 (5.45%)	2 (4.55%)	0.8374
Vomiting	3 (3.03%)	2 (3.64%)	1 (2.27%)	0.6941
Hepatoxicity Hypothyroidism Rash Radiation pneumonia	6 (6.06%) 6 (6.06%) 2 (2.02%) 2 (2.02%)	3 (5.45%) 4 (7.27%) 2 (3.64%) 2 (3.64%)	3 (6.82%) 2 (4.65%) 0 0	0.7775 0.5911 0.2013 0.2013

* *p* < 0.05.

## 4 DISCUSSION

SCLC is a highly aggressive malignancy associated with a bleak prognosis. This is particularly the case for ES‐SCLC, which carries a 5‐year OS rate of less than 7%.^7^, ^18^ Disease recurrence is the most prevalent cause of death among ES‐SCLC patients.^19^, ^20^, ^21^ Studies indicated that incorporating ICI into first‐line chemotherapy improves the median survival of ES‐SCLC patients.^9^, ^22^ While radiotherapy has demonstrated efficacy in enhancing local control and survival in ES‐SCLC, further exploration is necessary to uncover its potential synergistic effect with ICIs. The focus of this study was to explore the potential benefits of tRT for ES‐SCLC patients who underwent first‐line immunotherapy and chemotherapy. Results indicated a significant improvement in outcomes for patients who received tRT compared to those who did not in our patient cohort.

Chemotherapy has been the cornerstone of ES‐SCLC treatment for long, which a combination of etoposide with either cisplatin or carboplatin serve as the standard first‐line treatment regimen. Recently, ICIs targeting PD‐1 and PD‐L1 have shown promising clinical efficacy in SCLC treatment. The IMpower133 study, a phase III trial comparing the efficacy and safety of atezolizumab plus EC versus placebo plus EC as first‐line treatment for ES‐SCLC, reported a 2‐month median OS extension (12.3 months vs 10.3 months, *p* = 0.0154) and significantly improved 12‐month (51.9% vs 30.9%) and 18‐month (34.0% vs 21.0%) OS rates with atezolizumab plus EC. Furthermore, the median PFS was extended from 4.3 to 5.2 months, a 23% reduction in disease progression risk. The incidence of severe (grade 3/4) AEs was comparable between both groups.^8^ The CASPIAN study, which explored the use of the PD‐L1 inhibitor durvalumab combined with chemotherapy as first‐line treatment for ES‐SCLC, revealed that the durvalumab plus EC/EP group exhibited a significantly longer median OS than the chemotherapy group (13.0 months vs 10.3 months, *p* = 0.0047), with a 27% risk reduction of death (HR = 0.73, 95%CI 0.59–0.91). The incidence of AEs was comparable between the two groups (98.1% vs 97%).^9^ Notably, neither study permitted consolidation tRT.

Studies indicated that approximately 75% of patients have residual intrathoracic disease following first‐line treatment, and nearly 90% of patients experience intrathoracic disease progression within 1 year of treatment initiation.^21^, ^23^ Multiple studies attested to the positive response of SCLC to radiotherapy, with most patients achieving clinical respite through combined local radiotherapy.^12^, ^14^, ^24^ A phase III randomized controlled trial conducted by Slotman et al. in 2015 demonstrated that administering moderate‐dose tRT to patients with chemotherapy‐sensitive ES‐SCLC resulted in an approximately 50% reduction in the risk of thoracic relapse. Furthermore, this treatment approach was associated with an improved 2‐year OS (13% vs. 3%, *p* = 0.004) and PFS (24% vs. 7%, *p* = 0.001) compared to the control group.^14^

Studies suggest that current ICI therapy combined with RT may synergistically enhance anti‐tumor efficacy. RT is considered as a potential trigger for systemic anti‐tumor immune responses.^11^ The efficacy and safety of this combination have been explored in LS‐SCLC^25^ and preliminarily confirmed in NSCLC^26^, ^27^ and metastatic solid tumors.^28^ Despite the individual benefits of immunotherapy and tRT for patients with ES‐SCLC, limited clinical trials have investigated the combination of tRT and immunotherapy. In the last 2 years, some pilot clinical trials and real‐world studies have examined the outcomes of ES‐SCLC patients receiving a combination of immunotherapy and tRT.^29^, ^30^, ^31^ A single‐arm phase I clinical trial conducted by James et al. in 2019 aimed to assess the efficacy and safety of combining pembrolizumab with tRT in patients with ES‐SCLC following induction chemotherapy. The study demonstrated good tolerability of concurrent pembrolizumab‐tRT treatment, with a negligible number of high‐grade AEs observed in the short‐term. Nonetheless, due to heterogeneous eligibility criteria, the PFS and OS rates were challenging to interpret.^30^ A retrospective study from Canada carried out a subgroup analysis on patients with ES‐SCLC who received first‐line treatment with atezolizumab plus EP chemotherapy. The study found that patients who received tRT had a median PFS of 12.5 months, in contrast to 5.8 months for those who did not receive tRT. Moreover, the tRT group's median OS was not reached, while the non‐tRT group had a median OS of 11.5 months. Multivariate analysis revealed that tRT was significantly associated with improved OS. Notably, the study also found that tRT did not increase the risk of AEs.^29^ A study from Taiwan retrospectively evaluated the effectiveness and safety of tRT in ES‐SCLC patients who received immunotherapy plus EP chemotherapy as first‐line treatment. The results indicated that adding tRT significantly improved OS compared to those who did not receive tRT (not reached vs. 9.6 months, 95% CI 2.5–16.6).^32^ Another preclinical study and retrospective analysis from Southern Italy demonstrated that consolidation tRT was associated with significantly longer PFS rates at 1 year (61% vs. 31%, respectively, *p* < 0.001) and a trend towards improved OS rates at 1 year (80% vs. 61%, *p* = 0.027) compared to systemic therapy alone.^33^ Collectively, these findings suggest that incorporating tRT into the treatment regimen for ES‐SCLC patients receiving immunotherapy plus chemotherapy may be a promising strategy to enhance clinical outcomes.

Our study findings align with previous research, demonstrating that the incorporation of tRT following first‐line chemotherapy and immunotherapy can notably enhance OS and PFS in patients with ES‐SCLC. The AEs associated with this combination were manageable. Further, subgroup analysis solidified the survival benefits offered by tRT. In addition, our study also found that patients with liver metastases had a worse prognosis, which is consistent with the results of the secondary analysis of the CREST phase III trial.^34^ Notably, in our study, liver metastases were identified as an independent prognostic factor affecting prognosis in patients with ES‐SCLC, highlighting the importance of tailoring treatment to this subgroup.

It is also noteworthy that the TREASURE study,^35^ which aimed to evaluate tRT (30 Gy/10 f) concurrently with maintenance atezolizumab following standard first‐line atezolizumab plus etoposide‐cisplatin chemotherapy in patients with ES‐SCLC who achieved remission or stable disease, was terminated due to unexpected safety concerns, specifically a significant increase in toxicity. In our retrospective study, there was no statistically significant difference in the incidence of grade 3–4 adverse events between the two cohorts, suggesting that tRT did not lead to a notable increase in safety toxicity in our patient population. Nevertheless, being a retrospective study, it may have suffered from selection bias and was unable to control for all variables that may affect treatment outcomes. Additionally, our study design lacked randomization, which may limit the generalizability of our findings. Nonetheless, our results offer valuable insights into the potential efficacy of tRT in ES‐SCLC and underscore the need for further prospective studies to confirm these findings and elucidate the optimal therapeutic strategy for this patient population. The contrasting results between our study and the TREASURE trial highlight the complexity of ES‐SCLC treatment and the importance of carefully designed clinical trials in guiding clinical practice.

Based on limited clinical data on radiotherapy combined with immunotherapy for ES‐SCLC, the optimal dose for tRT remains controversial and requires further investigation. A retrospective study from the National Cancer Database analyzing chest consolidation radiotherapy for ES‐SCLC showed that increasing the dose to at least 45 Gy was an independent predictor of improved survival, significantly enhancing OS compared with lower doses.^36^ Conversely, another retrospective study showed that tRT at 45 Gy/30 f had a better survival benefit than tRT at 60 Gy/30 f.^37^ The MATCH study (NCT04622228), a single‐arm Phase 2 trial, was designed to study the benefit of adding low‐dose radiation therapy (LDRT) (15 Gy/5 f) to atezolizumab combination chemotherapy as first‐line treatment for ES‐SCLC. As of the completion of the Phase 1 study, 21 patients were available for evaluation with a confirmed objective remission rate (ORR) of 95.2%, demonstrating impressive anti‐tumor activity, potential survival benefits, and good tolerability.^38^ LDRT, while providing limited cytotoxicity due to the lower dose, effectively leverages the high sensitivity of SCLC to radiation. This contributes to its effectiveness and offers new insights into the immunotherapy of “cold” tumors like SCLC by reprogramming the tumor microenvironment (TME) and providing better immunostimulatory modulation than conventional or high‐dose fractionated radiotherapy. This dual benefit of LDRT enhances both direct tumor control and the efficacy of concurrent immunotherapy.^16^ Given the varying doses reported in different studies and the potential benefits observed with both low and high doses, our study divided patients into two groups based on their radiotherapy dose: those who received less than 30Gy and those who received 30Gy or more. This cutoff was chosen to distinguish between lower doses often used for palliative purposes, aimed at symptom relief and local control, and higher doses typically used for consolidation or curative intent following chemotherapy. However, our study found no significant correlation between radiation dose and patient prognosis, suggesting that factors other than dose may play a more critical role in determining outcomes in ES‐SCLC. To validate these findings and further investigate the effects of different dosages and regimens on patient prognosis, more prospective randomized controlled trials are warranted.

Compared to previous retrospective studies, our research presents several advantages. First, we incorporated a larger sample size. Futhermore, through subgroup analysis, we further validated the therapeutic impact of tRT. Finally, we documented AEs with greater detail. However, as a retrospective study, there are inevitable limitations. First of all, due to the lack of random allocation to treatment groups, there is a risk of selection bias potentially leading to inaccuracies in the results. Second, data collection from a single center and the relatively small sample size may limit the generalizability of the findings to other population groups or settings.

## 5 CONCLUSION

Our study suggested that tRT could prove beneficial for patients with ES‐SCLC undergoing first‐line immunotherapy and chemotherapy. Further prospective investigation is necessary to confirm and more thoroughly understand its potential benefits and risks.

## AUTHOR CONTRIBUTIONS

**Xiaoli Mu:** Conceptualization (equal); formal analysis (equal); investigation (equal); writing – original draft (equal). **Yixin Zhou:** Conceptualization (equal); data curation (equal); visualization (equal). **Qing Liu:** Data curation (equal); investigation (equal); validation (equal). **Jiantao Wang:** Data curation (equal); formal analysis (equal); resources (equal); visualization (equal). **Feng Xu:** Project administration (equal); supervision (equal). **Feng Luo:** Investigation (equal); resources (equal). **Ke Wang:** Data curation (equal); formal analysis (equal). **Lu Li:** Resources (equal); validation (equal). **Panwen Tian:** Supervision (equal); visualization (equal). **Yalun Li:** Methodology (equal); software (equal). **Jiewei Liu:** Data curation (equal); validation (equal). **Yan Zhang:** Conceptualization (equal); visualization (equal). **Jiyan Liu:** Methodology (equal). **Yan Li:** Conceptualization (equal); resources (equal).

## FUNDING INFORMATION

The work was supported by the West China Hospital of Sichuan University 2023 Clinical Research Incubation Project (2023HXFH020), Sichuan Province Science and Technology Support Program (2024YFHZ0012, Clinical and Translational Medicine Research Special Programme, Chinese Academy of Medical Sciences (2022‐I2M‐C&T‐B‐106) and 2022 Wu Jieping Medical Foundation (HX‐H2212352).The funders had no role in study design, data collection and analysis, decision to publish, or preparation of the manuscript.

## CONFLICT OF INTEREST STATEMENT

The authors have no conflict of interest.

## ETHICS STATEMENT

Institutional Review Board Statement: This study was approved by the Institutional Review Board of West China hospital of Sichuan University (No.2023‐1547). Informed Consent: According to the regulator, informed consent can be waived for a non‐interventional study. Registry and the Registration No. of the study/trial: N/A. Animal Studies: N/A.

## Data Availability

The datasets presented in this article are not readily available because part of the data included in the study came from a single institution and was not publicly available.
